# Core–shell nanospheres behind the blue eyes of the bay scallop *Argopecten irradians*

**DOI:** 10.1098/rsif.2019.0383

**Published:** 2019-10-23

**Authors:** Olivia K. Harris, Alexandra C. N. Kingston, Caitlin S. Wolfe, Soumitra Ghoshroy, Sönke Johnsen, Daniel I. Speiser

**Affiliations:** 1 Department of Biological Sciences, University of South Carolina, Columbia, SC 29208, USA; 2 Electron Microscopy Center, University of South Carolina, Columbia, SC 29208, USA; 3 Department of Biological Sciences, University of Cincinnati, Cincinnati, OH 45221, USA; 4 Department of Biology, Duke University, Durham, NC 27708, USA

**Keywords:** biophotonics, iridescence, optics, scattering, structural colour, visual ecology

## Abstract

The bay scallop *Argopecten irradians* (Mollusca: Bivalvia) has dozens of iridescent blue eyes that focus light using mirror-based optics. Here, we test the hypothesis that these eyes appear blue because of photonic nanostructures that preferentially scatter short-wavelength light. Using transmission electron microscopy, we found that the epithelial cells covering the eyes of *A. irradians* have three distinct layers: an outer layer of microvilli, a middle layer of random close-packed nanospheres and an inner layer of pigment granules. The nanospheres are approximately 180 nm in diameter and consist of electron-dense cores approximately 140 nm in diameter surrounded by less electron-dense shells 20 nm thick. They are packed at a volume density of approximately 60% and energy-dispersive X-ray spectroscopy indicates that they are not mineralized. Optical modelling revealed that the nanospheres are an ideal size for producing angle-weighted scattering that is bright and blue. A comparative perspective supports our hypothesis: epithelial cells from the black eyes of the sea scallop *Placopecten magellanicus* have an outer layer of microvilli and an inner layer of pigment granules but lack a layer of nanospheres between them. We speculate that light-scattering nanospheres help to prevent UV wavelengths from damaging the internal structures of the eyes of *A. irradians* and other blue-eyed scallops.

## 1. Background

Be they bright or dull, conspicuous or cryptic, the colours of animals are often of adaptive significance [1]. Animals produce colours primarily through the absorption of light by pigments, the scattering of light by nanoscale structures or interactions between the two [2,3]. The colour and intensity of light scattered by nanoscale structures depend on a variety of factors, including their shape, size, refractive index (RI), packing density and arrangement. Further, colours produced by ordered photonic nanostructures are often iridescent because the wavelengths they scatter towards a viewer depend on both the position of the viewer and the angle of incident light [4]. Recent efforts by researchers in fields from bio-engineering to evolutionary biology have sought to address how and why animals produce structural colours [1,5].

Like many other animals, such as arthropods (e.g. [6]) and vertebrates (e.g. [7]), molluscs produce colours, in part, through the coherent scattering of light by photonic nanostructures. In many cases, these nanostructures have linear dimensions of the order of a quarter wavelength of light (approx. 125 nm *in vacuo*), have a high RI and are embedded in a low-RI medium. Cephalopods, for example, have light-reflecting organs termed iridophores that scatter light using multilayer reflectors in which high-RI layers of protein approximately 100 nm thick alternate with low-RI layers of cytoplasm [8,9]. Among gastropods, the blue stripes in the shell of the blue-rayed limpet *Patella pellucida* are produced by multilayer reflectors in which the high-RI layers are 100 nm thick sheets of calcitic (CaCO_3_) shell material [10]. Among bivalves, the iridophores of the giant clam *Tridacna gigas* scatter light using multilayer reflectors with protein-based high-RI layers approximately 70 nm thick [11,12]. As another example, the ‘disco’ clam *Ctenoides ales* produces its ‘electric’ displays using random close-packed arrays of 300 nm diameter silica (SiO_2_) spheres that strongly scatter visible light [13].

**Figure 1.**
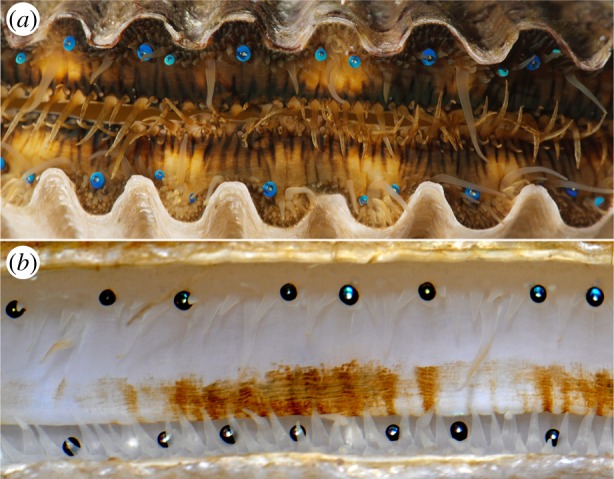
The blue-eyed bay scallop *A. irradians* (*a*) and the black-eyed sea scallop *P. magellanicus* (*b*). The blue hues associated with some of the eyes of *P. magellanicus* are due to the reflection of light by the mirror at the back of each eye. Photo credits: David Liittschwager (*a*) and Sönke Johnsen (*b*). (Online version in colour.)

The eyes of scallops, another type of bivalve mollusc (family Pectinidae), have also drawn attention for their use of photonic nanostructures. Unlike the eyes of most other animals, those of scallops focus light using a mirror [14]. The mirror in the scallop eye is a multilayer reflector in which guanine crystals form high-RI layers approximately 75 nm thick [15,16]. In some species of scallop, the external surfaces of the eyes are a bright, iridescent blue (figure 1*a*), which suggests that photonic nanostructures contribute to their appearance. Unlike the light-focusing mirror, however, the structural basis for the external coloration of scallop eyes is unknown.

Here, we use reflectance spectroscopy, transmission electron microscopy (TEM), energy-dispersive X-ray spectroscopy (EDS) and optical modelling to characterize the nanostructures underlying the iridescent blue appearance of the eyes of the bay scallop *Argopecten irradians* (figure 1*a*). For a comparative perspective, we also examine the black eyes of the sea scallop *Placopecten magellanicus* (figure 1*b*). By taking a mechanistic approach to the study of eye colour in scallops, we hope to gain further insights into the function and evolution of these unique mirror-based eyes.

## 2. Methods

### 2.1. Specimen collection and care

We obtained specimens of *A. irradians* from Gulf Specimen Marine Laboratory (Panacea, FL, USA) and those of *P. magellanicus* from Marine Biological Laboratory (Woods Hole, MA, USA). Prior to histological examination, we kept animals at the University of South Carolina (UofSC; Columbia, SC, USA) in a 150 gallon ‘Living Stream’ System (Frigid Units, Toledo, OH, USA) with recirculating seawater maintained at a temperature of 20°C and a salinity of 33 ppt. We provided light to these animals on a 12 L ∶ 12 D cycle using two Hydra TwentySix LED modules (AquaIllumination, Ames, IA, USA).

### 2.2. Reflectance spectrometry

We measured the spectral reflectance of the eyes of *A. irradians* using a modified Olympus CX-31 microscope (Center Valley, PA, USA). We replaced the right eyepiece of the microscope with a custom-made adapter to which we attached a Y-shaped reflection probe (QR400-7-UV-VIS; Ocean Optics, Dunedin, FL, USA) that supplied light from a 20 W tungsten halogen lamp with an emission range of 360–2400 nm (HL-2000-HP-FHSA; Ocean Optics) and carried reflected light back to a Flame-S-VIS-NIR-ES spectrometer (Ocean Optics) that we operated using Ocean View software (Ocean Optics). We focused light onto samples using an Olympus 10X PlanC N UIS2 objective. To standardize our measurements, we used a reflectance standard made of Spectralon (WS-1-SL; Ocean Optics). Because fixing the eyes of scallops appears to alter their colour slightly, we measured the spectral reflectance of fresh, unfixed eyes. We compared our reflectance measurements from the eyes of scallops with measurements taken from the unpigmented mantle tissue immediately adjacent to the eyes.

### 2.3. Transmission electron microscopy and energy-dispersive X-ray spectroscopy

For examination by TEM, we fixed eyes from *A. irradians* and *P. magellanicus* in a 2.5% solution of 0.1 M cacodylate-buffered glutaraldehyde (pH 7.4) overnight at room temperature. We then washed eyes five times in 0.1 M cacodylate buffer, post-fixed them in 1% osmium tetroxide (OsO_4_) for 1 h at 4°C and washed them several times in 0.1 M cacodylate buffer. Next, we dehydrated eyes in a graded series of EtOH solutions (50%, 70%, 80%, 95% and 100% twice) in which each step lasted 10 min. After dehydrating eyes, we incubated them at room temperature in a series of solutions that started with 100% acetone, moved to a series of mixtures of acetone and epoxy resin (EMBed 812; Electron Microscopy Sciences, Hatfield, PA, USA) and ended with 100% epoxy resin. We then transferred eyes to an embedding mould, added fresh epoxy resin and incubated them for 24 h at 60°C. From our cured blocks, we cut sections 60–80 nm thick using a Sorvall MT2B ultramicrotome fitted with a Microstar diamond knife (Huntsville, TX, USA). We cut sagittal sections from some eyes and coronal sections from others. We collected sections of eyes onto 300 mesh copper grids. To increase image contrast using TEM, we applied to these sections 5% uranyl acetate and lead citrate as electron-dense stains. We imaged sections at the Electron Microscopy Center at the University of South Carolina using either a Hitachi H8000 or HT7800 TEM (Hitachi High Technologies, Tokyo, Japan). To analyse the elemental composition of these sections, we used an EDS detector (Oxford Instruments, Concord, MA, USA) mounted to the Hitachi HT7800 TEM. We performed TEM imaging and EDS analysis at an operating voltage of 80 kV and a column vacuum pressure of 3.2 × 10^−0.5^ Pa.

### 2.4. Optical modelling

We measured the sizes of structures in our TEM images using ImageJ [17]. In our images, we noted the presence of core–shell nanospheres in the eyes of *A. irradians*. We estimated the average diameters of the cores and shells of these three-dimensional spheres by fitting the histograms of the diameters of 437 circular core sections and 393 circular shell sections to a model in which we assumed that the three-dimensional diameters of the cores and shells were distributed normally and intersected randomly by the planes of sectioning. We estimated the volume density of the nanospheres by counting the number of them present in 20 non-overlapping regions of our TEM images that were 2 × 2 µm in size and then used standard stereological methods to convert this value to a volume density. Next, we followed methods adapted from Bettelheim & Siew [18] and described in Dougherty *et al.* [13] to model the angle-weighted scattering of light by these nanospheres as a function of their diameter and packing density. In our optical model, we assumed that the nanospheres have a RI of either 1.44 or 1.55, spanning the range of estimates for the RI of the protein-based, high-RI layers in the light-reflecting organs of cephalopods [19,20]. We also assumed that the nanospheres are embedded in the cytoplasm, which has a RI of 1.35 [21].

## 3. Results

### 3.1. Reflectance spectrometry

**Figure 2.**
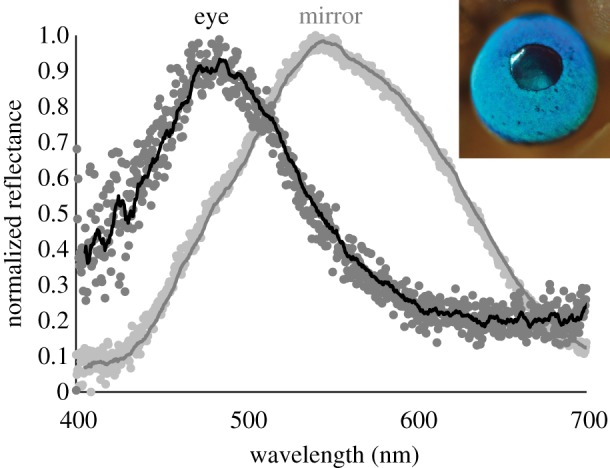
Normalized reflectance spectra of the external surface of the eye (*n* = 3) and the focusing mirror (*n* = 2) of the bay scallop *A. irradians*. The points represent data that we normalized and averaged for each tissue type, and the lines represent moving averages of 15 consecutive data points (equivalent to 5 nm). Inset: an iridescent blue eye from a living, intact specimen of *A. irradians*. Photo credit: David Liittschwager. (Online version in colour.)

Within the visible spectrum, reflectance spectrometry indicates that the external surfaces of the blue eyes of *A. irradians* have a peak reflectance at approximately 475 nm (figure 2). We also found that the mirror in the eye of *A. irradians* has a peak reflectance at approximately 540 nm, although this value is almost certainly angle-dependent and influenced by the absorption of light by the two retinas within the eye (figure 2).

### 3.2. Morphology

**Figure 3.**
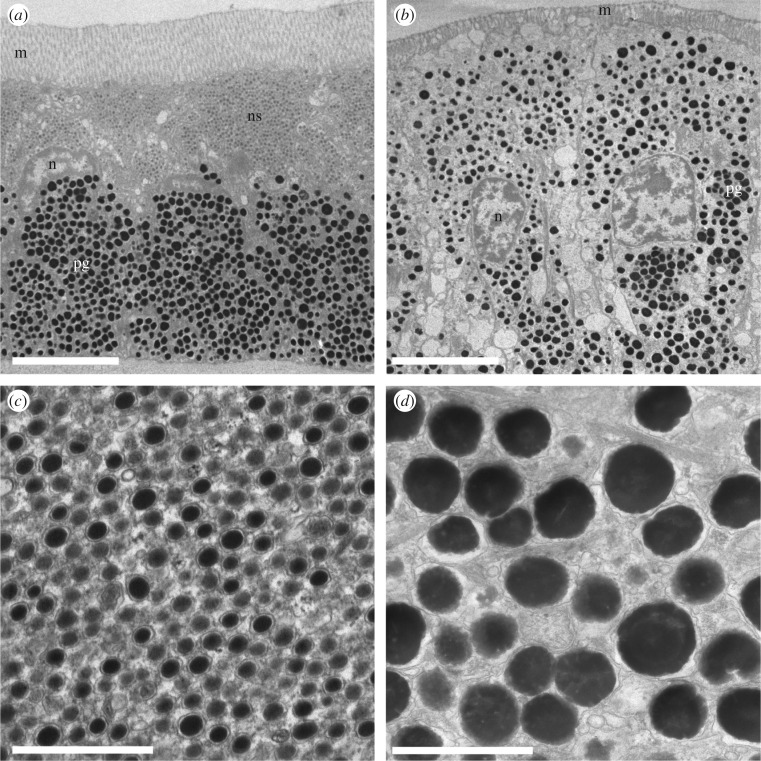
TEM images of pigmented epithelial cells from *A. irradians* (*a*) and *P. magellanicus* (*b*), along with higher-magnification images of photonic nanospheres (*c*) and pigment granules (*d*) from pigmented epithelial cells from the eyes of *A. irradians*. Scale bars, (*a*,*b*) 5 μm and (*c*,*d*) 1 μm. Abbreviations: m, microvilli; n, nucleus; ns, nanospheres; pg, pigment granules.

The external surfaces of the eyes of scallops are formed by a single, continuous layer of epithelial cells. Pigmented epithelial cells cover most of the eye and transparent cells comprise the cornea. In the blue-eyed *A. irradians*, the pigmented epithelial cells have three distinct layers: an outer layer of microvilli, a middle layer of nanospheres and an inner layer of pigment granules (figure 3*a*). In comparison, epithelial cells from the black-eyed *P. magellanicus* have an outer layer of microvilli and an inner layer of pigment granulates but lack a layer of nanospheres between them (figure 3*b*).

The epithelial cells covering the blue eyes of *A. irradians* are approximately 15 µm tall and 5 µm wide with microvilli approximately 3 µm long from tip to base and approximately 100 nm wide (figure 3*a*). We did not find morphological differences between the microvilli of the pigmented and non-pigmented epithelial cells. The nanospheres in the eyes of *A. irradians* have electron-dense cores surrounded by less electron-dense shells (figure 3*c*). We measured the diameters of the cores and shells of these nanospheres and found them to be 121 ± 16 nm (mean ± s.d.; *N* = 437) and 159 ± 20 nm (*N* = 393), respectively. From these measurements, we inferred that the nanospheres are approximately 180 nm in diameter and consist of electron-dense cores approximately 140 nm in diameter surrounded by less electron-dense shells 20 nm thick. The nanospheres appear to be random close-packed with a volume density of approximately 60% (figure 3*c*). In most cases, the nanospheres fill the distal half of a pigmented epithelial cell and are separated from the pigment granules in the proximal half of the cell by a crescent-shaped nucleus (figure 3*a*). Compared with the nanospheres, the pigment granules in the epithelial cells are more electron-dense, less tightly packed, larger (with diameters of 380 ± 57 nm; *N* = 27) and less regular in shape (figure 3*d*).

Epithelial cells from the black eyes of the sea scallop *P. magellanicus* appear to be slightly larger than those from the blue eyes of *A. irradians*, with heights and widths of approximately 18 µm and 6 µm, respectively (figure 3*b*). Their microvilli measure approximately 1 µm from tip to base, making them shorter than those observed in *A. irradians*. We did not observe morphological differences between the microvilli of the pigmented epithelial cells and those of the transparent epithelial cells of the cornea. The nuclei of the epithelial cells of the eyes of *P. magellanicus* are oval-shaped and positioned centrally. The pigment granules in the eyes of *P. magellanicus* fill the entire cytoplasmic space of the epithelial cells, are irregular in shape and size, and appear to be packed less densely than the pigment granules we observe in the eyes of *A. irradians*.

### 3.3. Energy-dispersive X-ray spectroscopy

**Figure 4.**
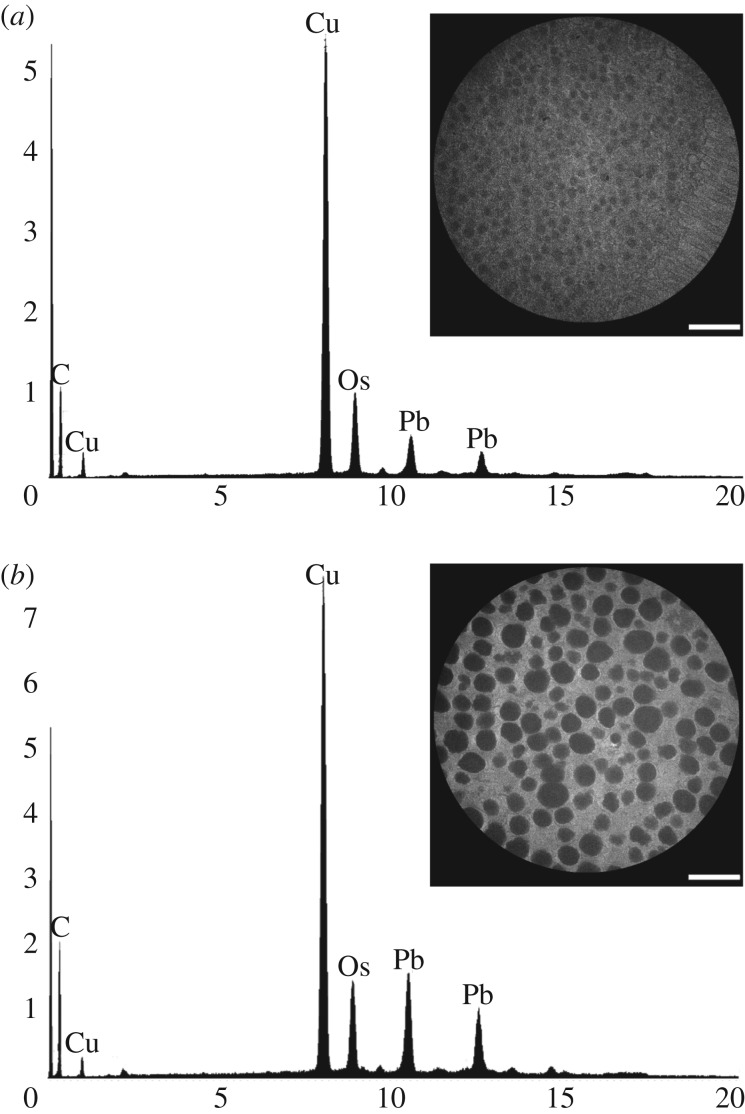
Elemental analysis using EDS shows the composition of the nanospheres (*a*) and pigment granules (*b*) we identified in pigmented epithelial cells from the eyes of *A. irradians*. Peaks for Cu and Pb are from the copper grids on which we mounted samples and a lead citrate stain, respectively. We do not see evidence of calcium or silicon in either set of structures, suggesting that they are not mineralized. Insets: the fields of our samples analysed by EDS. Scale bars, 1 µm.

Using EDS, we investigated the elemental compositions of the nanospheres (figure 4*a*) and pigment granules (figure 4*b*) in epithelial cells from the eyes of *A. irradians*. We did not detect above-background levels of calcium or silicon for either the nanospheres or the pigment granules, indicating it is unlikely either set of structures is composed of calcium carbonate (CaCO_3_) or silica (SiO_2_).

### 3.4. Optical modelling

**Figure 5.**
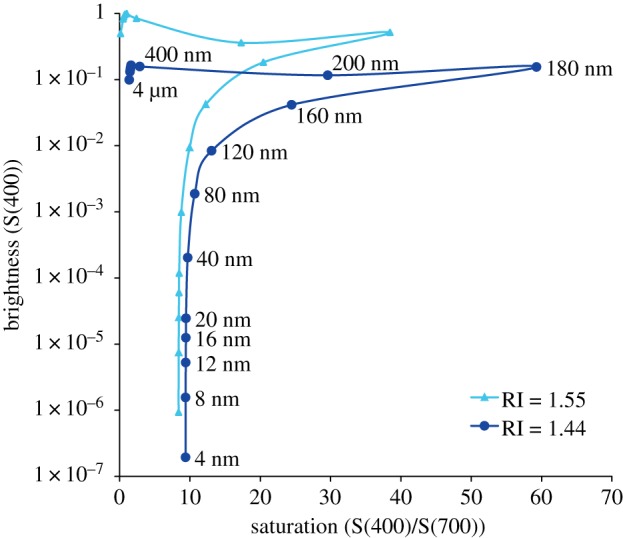
The effect of diameter on the brightness and colour saturation of light scattered by collections of random close-packed nanospheres with RIs of either 1.44 or 1.55. We assumed that these nanospheres are embedded in a medium with a RI of 1.35 (similar to that of cytoplasm). We defined brightness as the angle-weighted scattering at 400 nm (S(400)) and colour saturation as the ratio between the angle-weighted scattering at 400 nm and that at 700 nm (S(400)/S(700)). The data points for the higher-RI nanospheres correspond in order to the labelled data points for the lower-RI nanospheres. (Online version in colour.)

Our computational model indicates that random close-packed spheres with diameters of approximately 180 nm produce angle-weighted scattering that is both nearly maximal and maximally wavelength-dependent, i.e. both bright and blue (figure 5). Here, we defined brightness as the angle-weighted scattering at 400 nm and saturation as the ratio between the angle-weighted scattering at 400 nm and that at 700 nm. Using our model to explore the scattering of light by nanospheres of different sizes, we find that aggregations of small spheres (with diameters of the order of approx. 4 nm) scatter very little light. As spheres increase in diameter from 4 nm towards approximately 180 nm, the brightness and colour saturation of the light they scatter increase. As diameters increase past approximately 180 nm towards 4 µm, brightness changes little, but there is a rapid drop in the wavelength dependence of the scattering (figure 5). Our results were similar whether we modelled spheres with RIs of 1.44 or 1.55, although we did find that collections of nanospheres with higher RIs produced a brighter, less saturated blue than collections of nanospheres with lower RIs (figure 5).

## 4. Discussion

### 4.1. Photonic nanospheres contribute to the blue colour of the eyes of scallops

We find that random close-packed spheres maximally scatter bright blue light when their diameters are approximately 180 nm (figure 5), the same diameter as the nanospheres we identified in pigmented epithelial cells from the eyes of *A. irradians*. Thus, it is likely that the eyes of *A. irradians* gain their iridescent blue colour from the scattering of light by photonic nanospheres. The pigment granules underlying the nanospheres may further enhance the saturation of the colour of the eyes of *A. irradians* by absorbing longer-wavelength light that passes through the nanospheres without being scattered. In other examples of structural colour, absorbing pigments play a similar role in enhancing the saturation of colours produced by wavelength-dependent coherent scattering [2].

A comparative perspective supports our hypothesis that photonic nanospheres underlie the blue colour of the eyes of *A. irradians*: we find that epithelial cells from the black eyes of *P. magellanicus* lack the layer of nanospheres we observe in corresponding cells from the blue eyes of *A. irradians*. Furthermore, random close-packed nanospheres, similar in size and shape to those we observe in *A. irradians*, appear to be present in the pigmented epithelial cells surrounding the blue eyes of the lion's paw scallop *Nodipecten nodosus* [22].

Evidence suggests that the nanospheres in the eyes of *A. irradians* are composed of protein. It is unlikely that they are mineralized: an elemental analysis using EDS indicates that they do not contain detectable amounts of calcium or silicon (figure 4*a*). It is also unlikely that they are formed from pigment: images of sectioned scallop eyes obtained by light and confocal microscopy reveal that the distal halves of epithelial cells from the eyes of *A. irradians* (the location of the nanospheres) are transparent, whereas the proximal halves of these cells (the location of the pigment granules) are opaque [23,24]. Steps towards more fully evaluating the structural basis of eye colour in scallops will include characterizing the material composition of the nanospheres, measuring the RI (or RIs) of the nanospheres and identifying the pigment(s) housed in the pigment granules lying underneath the nanospheres.

### 4.2. Function and evolution of blue eyes in scallops

Molluscs use structural colours for a variety of purposes. Cephalopods, for example, use structural colours for tasks ranging from crypsis to interspecific communication [8]. Among bivalves, giant clams, such as *T. gigas*, may use their iridophores to scatter light throughout their mantle tissues to more evenly illuminate their photosynthetic endosymbionts [12]. A different type of bivalve, the ‘disco’ clam *C. ales*, has been hypothesized to use a dynamic visual display involving structural components as an aposematic signal [13]. It is well established that scallops use specular reflection by the mirror at the back of their eye for image formation [14–16,25]. However, the function of the iridescent blue epithelial cells surrounding the eyes of some scallops remains unclear.

Although bright blue, the eyes of scallops are small enough (less than 1 mm in diameter) that it is unlikely most viewers (such as predators) would be able to distinguish them at ecologically relevant distances. Thus, we find it unlikely that eye colour has a role in crypsis or visually mediated communication in scallops. Instead, we speculate that the eyes of some scallops are blue because an aggregation of non-absorbing nanospheres that maximally scatters blue light will scatter UV radiation even more strongly. Preventing UV wavelengths from penetrating their eyes may be a particular concern for scallops because their retinas do not contain the UV-absorbing screening pigments that are associated with the photoreceptors of most camera-type and compound eyes [26]. By scattering away UV wavelengths rather than absorbing them, scallops may slow the rate at which the screening pigments of their eyes degrade due to photo-oxidation [27].

If the eyes of scallops protect their internal structures by scattering UV wavelengths from their external surfaces, we may expect shallow-dwelling scallops to be more likely to have blue eyes than deeper-dwelling species. Indeed, this hypothesis holds for the species we study here: the blue-eyed *A. irradians* tends to live in brighter, shallower water than the black-eyed *P. magellanicus* [28]. However, eye colour varies beyond blue and black in scallops: some species, like the saucer scallop *Amusium balloti*, have brown eyes [24] and others, like the coral-boring scallop *Pedum spondyloideum*, have red eyes (S.J. 2008, personal observation). A study of eye colour in scallops, taking note of phylogenetic relationships (e.g. [29]) and depth range, may help shed further light on whether structural colour is an adaptive or non-adaptive feature of the eyes of these bivalves.

## 5. Conclusion

As their diameters increase through three orders of magnitude (4 nm–4 µm), we find that collections of random, close-packed, non-absorbing spheres will first appear clear, then a bright and highly saturated blue, then a bright and less saturated blue and finally a bright white. With diameters of approximately 180 nm, the nanospheres in the eyes of *A. irradians* are an ideal size for producing a bright blue coloration. It is unclear, however, whether blue eyes are an adaptive feature of scallops. We suggest, tentatively, that a bright blue surface may help shallow-dwelling scallops minimize the intrusion of UV wavelengths into their eyes. From the perspective of biologically inspired design, the core–shell structure of the nanospheres we identified in *A. irradians* is intriguing for several reasons: first, evidence suggests that nanospheres with high-RI cores and low-RI shells produce brighter colours than nanospheres with a homogeneous RI; and second, the colours of core–shell nanospheres may be tuned by altering the thickness of their low-RI shell [30]. A broader comparative study of the structural colours of scallop eyes may provide further biological insights into the engineering of colour.

## Data accessibility

This article has no additional data.
